# Diagnostic Utility of Broad Range Bacterial 16S rRNA Gene PCR with Degradation of Human and Free Bacterial DNA in Bloodstream Infection Is More Sensitive Than an In-House Developed PCR without Degradation of Human and Free Bacterial DNA

**DOI:** 10.1155/2014/108592

**Published:** 2014-07-09

**Authors:** Petra Rogina, Miha Skvarc, David Stubljar, Romina Kofol, Achim Kaasch

**Affiliations:** ^1^Novo Mesto General Hospital, 8000 Novo Mesto, Slovenia; ^2^Institute of Microbiology and Immunology, Faculty of Medicine, 1000 Ljubljana, Slovenia; ^3^Institute for Medical Microbiology, Immunology and Hygiene, University of Cologne, Cologne 50935, Germany

## Abstract

We compared a commercial broad range 16S rRNA gene PCR assay (SepsiTest) to an in-house developed assay (IHP). We assessed whether CD64 index, a biomarker of bacterial infection, can be used to exclude patients with a low probability of systemic bacterial infection. From January to March 2010, 23 patients with suspected sepsis were enrolled. CD64 index, procalcitonin, and C-reactive protein were measured on admission. Broad range 16S rRNA gene PCR was performed from whole blood (SepsiTest) or blood plasma (IHP) and compared to blood culture results. Blood samples spiked with *Staphylococcus aureus* were used to assess sensitivity of the molecular assays *in vitro*. CD64 index was lower in patients where possible sepsis was excluded than in patients with microbiologically confirmed sepsis (*P* = 0.004). SepsiTest identified more relevant pathogens than blood cultures (*P* = 0.008); in three patients (13%) results from blood culture and SepsiTest were congruent, whereas in four cases (17.4%) relevant pathogens were detected by SepsiTest only. *In vitro* spiking experiments suggested equal sensitivity of SepsiTest and IHP. A diagnostic algorithm using CD64 index as a decision maker to perform SepsiTest shows improved detection of pathogens in patients with suspected blood stream infection and may enable earlier targeted antibiotic therapy.

## 1. Background

Sepsis affects millions of people around the globe each year and is a major cause of death [1, 2]. It is defined as a systemic inflammatory response syndrome (SIRS) that is caused by an infection [3]. The standard approach to detecting the underlying infection is by culturing blood and other specimens. Blood cultures can detect a wide range of microorganisms and are well established in the diagnostic pathway. However, shortcomings do exist. For example, blood cultures only detect viable organisms that are able to grow in the blood culture medium and often remain negative in patients that have previously received antimicrobial therapy. Another criticism is that the final results from blood cultures are available too late and are thus of limited use for guiding initial therapy [4]. In the past few years, molecular methods of detecting microorganisms have been developed that are based on the polymerase chain reaction (PCR). In general these methods offer a shorter time to result, but they are more expensive, require highly trained staff, and have not been evaluated well in the clinical context [5]. However, they might be well suited to complement the standard diagnostic approach.

A sensitive and specific way to detect bacteria or fungi directly from blood is based on amplifying the small subunit (16S and 18S) ribosomal RNA (rRNA) genes by PCR and subsequent sequence comparison to a database of known sequences [6]. Since rRNA genes are universally present, all microorganisms can be identified at least to genus level. Based on this principle, several methods have been developed, including the commercially available SepsiTest (ST, Molzym, Germany). Some protocols rely on detecting microorganisms in blood culture or in serum/plasma [7–9], whereas in others, including ST, DNA is isolated from whole blood [10]. Since whole blood contains more human DNA, an additional DNA degradation step is required to reduce the background signal which is caused by unspecific binding of amplification primers to human DNA [11, 12].

These molecular techniques have not reached clinical practice. The main reason is the associated cost. Therefore, samples need to be carefully selected to maximize clinical impact; that is, samples from patients with a low likelihood of systemic infection should be excluded. To identify suitable samples, clinical criteria (e.g., SIRS criteria) should be complemented by inflammatory biomarkers such as C-reactive protein (CRP) and procalcitonin (PCT) [13]. A novel and promising biomarker to predict severe systemic bacterial infection is CD64 expression on neutrophils [14], which showed a pooled sensitivity of 79% (95%, confidence interval (CI) 70% to 86%) and a specificity of 91% (95%, CI 85% to 95%) in a meta-analysis [15].

Currently, it is unclear whether broad range 16S rRNA gene PCR assays should be performed on plasma or whole blood. In this pilot study, we assessed two different molecular methods, ST and IHP, in clinical practice and compared the results to conventional blood culture. Furthermore, we tested whether inflammatory biomarkers may serve to exclude patients with a low probability of systemic bacterial infection.

## 2. Materials and Methods

### 2.1. Spiked Blood Samples

900 *μ*L of EDTA whole blood from healthy volunteers was spiked with 100 *μ*L of serial dilutions of* Staphylococcus aureus* (ATCC 29213) in sterile 0.9% NaCl with final concentrations ranging from 1.5 × 10^−2^ colony-forming units (CFU) per mL to 1.5 × 10^4^ CFU/mL. Concentrations above 1 CFU/mL were verified by overnight culture on blood agar plates at 37°C. To obtain plasma, spiked whole blood samples were centrifuged for 10 minutes at 800 ×g.

### 2.2. Clinical Study

The study was approved by the Slovenian National Medical Ethics Committee and informed consent was given by each enrolled patient. From January to March 2010, 23 consecutive adult patients of both sexes were enrolled in the study. They were admitted to the emergency department of a community secondary care hospital with 377 beds with clinical signs of severe infections with possible sepsis.

Clinical signs of possible bacterial infection were defined as the presence of at least two of the four criteria that define SIRS [3]: a body temperature above 38°C or below 36°C, a heart rate of more than 90 bpm, a respiratory rate of more than 20 breaths per minute or hyperventilation (PaCO_2_ less than 4.3 kPa), and leukocytosis (more than 12,000/mm^3^) or leukopenia (less than 4,000/mm^3^) or more than 10% premature granulocytes (band forms). Patients who had received antibiotic therapy for more than 24 hours before admission were excluded from the study protocol.

Blood samples for DNA isolation, CD64 expression, CRP (Siemens Health Care, Germany) and PCT (Brahms, Germany) levels, two sets of blood cultures (BACTEC plus aerobic/F and plus anaerobic/F, BD Biosciences, USA), and urine and respiratory tract cultures, as well as cultures from suspected infective foci, were obtained from all patients while they were still in the emergency department. Blood culture bottles were incubated for 5 days until they were considered negative (BACTEC 9240 System, BD Biosciences, USA). BC and other cultures were processed using standard microbiology culture methods.

Data on treatment and outcome was prospectively collected during the hospital stay by an infectious diseases physician. All cases were reviewed for plausibility by a clinical microbiologist.

### 2.3. CD64 Index Assay

The index of CD64 expression on neutrophils using 50 *μ*L of whole blood from of ethylenediaminetetraacetic acid (EDTA) tube was measured with the Leuko64 assay and calculated by Leuko64 QuantiCALC software (Trillium Diagnostic LCC, USA). As recommended by the manufacturer, a value greater than 1.2 was considered predictive for bacterial infection. The samples were processed immediately during workdays and within 24 hours during weekends.

### 2.4. Automated DNA Extraction and In-House Developed 16S rRNA PCR

All laboratory personnel involved in sample processing and analysis of nonculture based techniques were blinded with regard to results from conventional cultures. For IHP, automated DNA extraction was performed from 400 *μ*L of plasma or whole blood samples with the MagNA Pure Compact Nucleic Acid Isolation Kit on a MagNa Pure Compact Instrument (Roche Applied Science, Germany) using the Total NA Plasma 100–400 protocol. The final eluted volume was 100 *μ*L. Amplification of 16S rRNA gene was performed under the following in-house developed conditions on Thermocycler T3000 (Biometra, Germany): 2 min at 94°C, 35 cycles of 30 sec at 94°C, 30 sec at 59°C, 30 sec at 72°C, and 10 min at 72°C. To enhance amplification, two forward primers, 16SFa (5′-GCTCAGATTGAACGCTGG-3′), 16SFb (5′-GCTCAGGAYGAACGCTGG-3′), and one reverse primer, 16SR (5′-TACTGCTGCCTCCCGTA-3′), were used (TIB MOLBIOL, Germany). The PCR reaction contained 5 *μ*L of DNA sample and 0.4 *μ*L of 2 U/*μ*L EUB DNA polymerase (Minerva Biolabs, Germany) in final volume of 50 *μ*L.

The length of the amplified DNA product made using IHA (approx. 320 bp) was confirmed with 2% agarose gel electrophoresis. PCR product was purified with the Wizard SV Gel and PCR Clean-Up System (Promega, USA). Sequencing was performed on an ABI PRISM 310 Genetic Analyzer (Applied Biosystem, USA). The identity of amplified 16S rRNA sequences was determined using the basic local alignment search tool (BLAST) web based program (National Library of Medicine NCBI, USA) and the proprietary database SepsiTest BLAST (Molzym, Germany). Identification was considered sufficient when a similarity of more than 97% was met with a minimum read length of 250 base pairs.

### 2.5. Manual DNA Extraction and Broad-Range PCR with ST

Manual extraction from whole blood and PCR amplification was carried out with ST from 1 mL of* S. aureus *spiked whole blood or from 1 mL of a patient's whole blood. The kit includes a protocol for the lysis of human cells and degradation of human DNA and free bacterial DNA by a DNase. Pathogenic cells are then concentrated from lysate and treated with two reagents (BugLysis and *β*-mercaptoethanol) that hydrolyse the cell walls of bacteria and fungi. Pathogenic DNA is then bound, washed, and eluted into 100 *μ*L. Clinical samples were processed in duplicate to increase sensitivity as suggested by the manufacturer. All amplicons (approximately 450 bp long) were purified as described above and sequenced using the sequencing primers (SeqGP16: Gram-positive bacteria and SeqGN16: Gram-negative bacteria) supplied in the SepsiTest kit. Identification of pathogens was performed using the online search BLAST tool as described above. All samples were processed immediately during the working hours of our laboratory. Delays occurred during weekends because the sequencing services were inaccessible.

### 2.6. Statistical Analysis

Statistical analysis was performed by using Statistical Package for the Social Sciences 19.0 (SPSS, USA). Data was summarized in counts and percentage. A nonparametric Kruskal-Wallis test and Student's* t*-test were used to compare quantitative variables between four groups of SIRS diagnoses and between two groups of patients (patients with bacterial infection and patients without bacterial infection). Chi-square test was used to compare qualitative values. Area under the curve (AUC), sensitivity, and specificity were calculated.* P* values below 0.05 were set as statistically significant.

## 3. Results

*Staphylococcus aureus* was detected in spiked blood samples by both methods: automated bacterial DNA isolation followed by an in-house developed broad range 16S rRNA gene PCR assay, as well as with manual extraction using the ST protocol (Table 1). When strong bands were demonstrated in gel electrophoresis, 16S rRNA gene sequence analysis yielded reproducible results. When weak bands were present, pathogens could only be identified to genus level. The most sensitive detection (1.5 CFU/mL) was achieved by using ST on whole blood and IHP from plasma (Table 1). Both methods were further evaluated using clinical samples.

**Figure 1:**
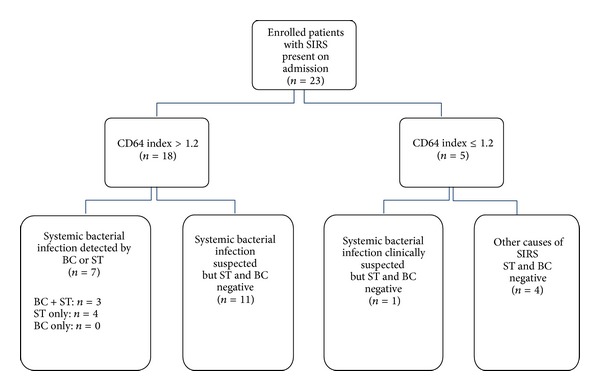
Flow diagram of patients enrolled in the study and results from CD64 index, BC, and ST (SIRS = systemic inflammatory response syndrome, ST = SepsiTest, and BC = blood culture).

In the clinical study, 61 patients were screened for SIRS criteria in the emergency department. 23 of the patients met the inclusion criteria and were enrolled in the study (Figure 1). All patients were subsequently transferred to an intensive care unit for supportive treatment. Enrolled patients were between 24 and 88 years old (median age 59 years). Systemic bacterial infection was suspected in 19 patients (82.6%); four patients (17.4%) with other reasons for SIRS were identified: three patients suffered from a myocardial infarct and one patient from thyrotoxicosis. All but one of the patients with suspected systemic bacterial infection showed an elevated CD64 index (Figure 1).

The clinical diagnosis of systemic bacterial infection was supported in seven patients (30.4%) by isolation/detection of plausible pathogens (Table 2). In three of these patients (13% of enrolled patients) BC yielded the same organism as was detected by ST, whereas in four patients (17.4%) a plausible causative organism was only found by ST (two of which were also detected by IHP). Three of these patients presented with pulmonary symptoms and* Streptococcus pneumoniae* was detected; in the other patient* Streptococcus salivarius* was detected as a plausible cause for infective endocarditis. In two further patients with suspected bacterial infection (8.7% of enrolled patients), ST detected* Streptococcus mitis *and* Staphylococcus hominis* which were considered contaminants on clinical grounds. Overall, significantly more plausible causative organisms were found by ST than by BC (*P* = 0.008).

IHP performed less reliably than ST. In two patients, the use of IHP from plasma failed to identify* Staphylococcus aureus*. On the other hand, IHP did not detect the two pathogens that were considered contaminants (Table 2).

In three patients with negative blood cultures and negative results of molecular methods, potential pathogens were found in other specimens:* Escherichia coli* isolated from urine and* Staphylococcus aureus* and* Streptococcus pneumoniae* isolated from the respiratory tract. However, it remained uncertain whether these pathogens were the cause of systemic infection.

When suspected contamination was not considered, BC and ST yielded concordant results in 19 (83%) patients, BC and IHP in 19 (83%) patients, and ST and IHP in 17 (74%) patients. However, ST was more sensitive than BC and IHP when measured against a constructed gold standard taking into account all available information. Plausible pathogens were detected in seven (30%) patients, whereas by BC and IHP plausible pathogens were detected in three (13%) patients each. Test performance measures are shown in Table 3.

The time to result for ST and IHP was approximately 10 hours during workdays and 24 hours during weekends. BC became positive after an average of 12 hours and species identification was performed after overnight incubation, typically yielding a result for identification after 24–48 h.

Whether the selected inflammatory biomarkers can predict systemic bacterial infection was assessed (Table 4). CD64 index was significantly higher in patients with documented systemic bacterial infection compared to patients where a bacterial infection was excluded (*P* = 0.0006); four out of five patients with a CD64 index below 1.2 did have an alternative cause of SIRS. The one remaining patient showed local infection (cellulitis of the calf), but relevant pathogens were not found. The biomarkers CRP and PCT did not show any statistically significant association with bacterial infection (*P* = 0.27 and *P* = 0.21, resp.).

## 4. Discussion

In a pilot study on 23 consecutive patients with SIRS, we compared a diagnostic workup with conventional microbiological techniques to two fairly novel diagnostic strategies based on broad range 16S rRNA gene PCR using whole blood or blood plasma. The use of molecular techniques resulted in improved detection of microorganisms causing bloodstream infection: by blood culture a causative microorganism was found in three (13%) patients, whereas molecular techniques detected plausible microorganisms in seven cases (30.4%). The rate of positive blood cultures in this study was within the expected range, including a recent study from Slovenia [16–19].

Molecular methods need specialized personnel and are cost-intensive. Diagnostic pathways that integrate molecular methods in routine care are lacking. Therefore, we assessed whether inflammatory biomarkers can be used to rule out systemic bacterial infection and thus could help to restrict molecular methods to patients with a high probability of systemic bacterial infection. The inflammatory marker CD64 index was more sensitive and more specific for systemic bacterial infection than CRP and PCT. Due to its high negative predictive value, CD64 index may be used to guide 16S rRNA gene PCR based diagnostics.

In this study molecular techniques were more sensitive in detecting microorganisms than blood culture. Other studies have reported the sensitivity of 16S rRNA gene PCR to be comparable to blood cultures [5, 20]. In the largest study using ST so far, 25 of 187 patients (13%) had relevant organisms identified by ST that did not grow in blood culture, but ST did not find all relevant organisms that grew in blood culture [21]. Therefore, it was suggested to use ST as an add-on to conventional microbiology.

Interpretation of studies that evaluate PCR-based methodology is hampered by the lack of a gold standard. On one hand, conventional culture may underestimate the presence of bacteria due to growth inhibition by antimicrobials. On the other hand, conventional culture is prone to contamination by skin flora and may thus overestimate the rate of bacteremia. In a recent study, the rate of true positive blood cultures ranged from 9.8% to 12.8% and the contamination rate ranged from 2.2% to 5.4% [18]. The final diagnosis of a possible bloodstream infection needs to be based on careful clinical evaluation by an infectious diseases physician, a laboratory, and microbiology data [22].

Molecular methods may also be compromised by contamination, either by laboratory contamination or by introducing skin flora during venipuncture [23]. To compensate for the lack of a clear gold standard, we constructed a gold standard from all available data, taking into account the clinical plausibility of detected pathogens. For example, in two cases from our study, ST identified organisms that were considered contamination on clinical grounds. In another patient with a history of intravenous drug abuse and infective endocarditis, the possible pathogen* Streptococcus salivarius* was identified by ST. Although conventional culture did not find any pathogen, the clinical condition of the patient improved after 6 weeks of antibiotic therapy. Therefore,* S. salivarius* was classified as causative pathogen. However, some uncertainty remains.

A comparison of the two molecular methods showed differences in sensitivity. Although* in vitro* experiments suggested a similar sensitivity of IHP from blood plasma and ST from whole blood, IHP did not detect relevant pathogens in four patients that were detected by ST. The techniques differ in a DNA degradation step that serves to degrade contaminating human DNA in the ST protocol. Although there is less human DNA present in blood plasma than in whole blood, it may be enough to disturb the assay. This also explains why the sensitivity of IHP from whole blood was low in the* in vitro* experiments. Removal of human DNA may thus be a critical step towards higher sensitivity [5, 20].

The pilot study has several limitations with the main limitation being its small size. Furthermore, the patients studied were a selected population and results may not therefore be generalizable. All patients presented with SIRS to the emergency department and were later admitted to an intensive care unit. Patients treated with antimicrobial therapy for more than 24 h were excluded. Another limiting factor of 16S rRNA gene PCR (and the CD64 index) is that yeast infections are not detected. In our patient population yeast infections were unlikely to occur, since the patients presented with community acquired infections and were not severely immunosuppressed. In principle, yeast could be detected by using similar techniques, for example, 18S rRNA gene sequencing and (1→3)*β*-D-Glucan as a biomarker [24].

Currently, molecular methods are unlikely to replace conventional blood cultures: they are resource-intensive, need specialized personnel, and have a higher hands-on time. Most importantly, they are currently not able to provide data on antibiotic susceptibility.

The strength of molecular methods lies within the higher sensitivity and the shorter time to result that can translate into earlier targeted antibiotic therapy. In our study, identification of microorganisms by molecular methods was achieved within 10 h during workdays, whereas conventional microbiology took at least 24 h. However, rapid diagnostic methods (e.g., using MALDI-TOF-MS) have been developed recently that considerably shorten the time to identification from blood cultures [25]. The higher sensitivity of molecular methods, however, makes them a useful addendum to conventional blood culture and further development can be expected with the future integration of next-generation sequencing.

## 5. Conclusion

In our pilot study, a diagnostic algorithm using CD64 index as a decision maker to perform 16S rRNA gene sequence analysis showed improved detection of pathogens in patients with suspected blood stream infection. For the tested molecular methods ST from whole blood was more sensitive than IHP from blood plasma. As a decision maker CD64 index discriminated better between patients with systemic bacterial infection and other causes of SIRS than PCT and CRP. These results offer an interesting perspective but need to be evaluated in larger clinical studies.

## Figures and Tables

**Table 1:** Sensitivity of different detection methods in whole blood and plasma measured in samples spiked with *S. aureus*. IHP from plasma and ST from whole blood showed equal sensitivity. Reported is the strength of visible bands in gel electrophoresis (“+” strong band present, “+/−” weak band present, and “−” no band present). The identity of bands was confirmed by sequencing (ST = SepsiTest, IHP = in-house PCR).

Final concentration of *S. aureus* (CFU/mL)	IHP from whole blood	IHP from plasma	ST from whole blood
0.015	−	−	−
0.15	−	−	−
0.75	−	−	+/−
1.5	−	+	+
1.5 × 10^1^	−	+	+
1.5 × 10^2^	−	+	+
1.5 × 10^3^	+/−	+	+
1.5 × 10^4^	+	+	+

**Table 2:** Patients with positive BC, ST, or IHP. Bacterial infection was considered confirmed when plausible pathogens were found in blood culture and by molecular methods.

Case	Comorbidities	Conventional microbiology	ST	IHP	Bacterial infection
1	Acute cholecystitis	*Klebsiella pneumoniae* (blood culture and respiratory tract)	*Klebsiella pneumoniae *	*Klebsiella pneumonia *	Confirmed

2	Alcoholic liver cirrhosis	*Staphylococcus aureus* (blood culture)	*Staphylococcus aureus *	Negative	Confirmed

3	Knee prosthesis	*Staphylococcus aureus* (blood culture and knee joint aspirate)	*Staphylococcus aureus *	Negative	Confirmed

4	Congestive heart failure, generalized atherosclerosis	Negative	*Streptococcus pneumoniae *	*Streptococcus pneumoniae *	Plausible pathogen

5	Congestive heart failure, diabetes type II	Negative	*Streptococcus pneumoniae *	*Streptococcus pneumoniae *	Plausible pathogen

6	Diabetes type II, chronic obstructive pulmonary disease	Negative	*Streptococcus pneumoniae *	Negative	Plausible pathogen

7	Injection drug use, endocarditis	Negative	*Streptococcus salivarius *	Negative	Plausible pathogen

8	Chronic renal failure, diabetes mellitus	Negative	*Staphylococcus hominis *	Negative	Considered contamination

9	Colon cancer	Negative	*Streptococcus mitis group *	Negative	Considered contamination

**Table 3:** Measures of test performance for BC, ST, and IHP against a constructed gold standard. PPV = positive predictive value, NPV = negative predictive value, ST = SepsiTest, IHP = in-house PCR, and BC = blood cultures.

	Sensitivity (%)	Specificity (%)	PPV (%)	NPV (%)
ST	52.6	100	100	30.8
IHP	15.8	100	100	20
BC	15.8	100	100	20

**Table 4:** Levels of inflammatory biomarkers in patients with and without systemic infection expressed as mean ± standard deviation (SD).

	CD64 index ± SD	PCT [*µ*g/L] ± SD	CRP [mg/L] ± SD
Systemic bacterial infection (*n* = 19)	2.35 ± 1.27	12.19 ± 28.57	174.24 ± 106.40
Systemic bacterial infection excluded (*n* = 4)	0.81 ± 0.13	2.49 ± 2.38	90 ± 69.46
